# Commentary: The value of life course epidemiology in low- and middle-income countries: an ageing perspective

**DOI:** 10.1093/ije/dyw109

**Published:** 2016-10-06

**Authors:** Stephen M Tollman, Shane A Norris, Lisa F Berkman

**Affiliations:** 1 MRC/Wits Rural Public Health and Health Transitions Research Unit, School of Public Health, Faculty of Health Sciences, University of the Witwatersrand, Johannesburg, South Africa; 2 MRC/Wits Developmental Pathways for Health Research Unit, School of Clinical Medicine, Faculty of Health Sciences, University of the Witwatersrand, Johannesburg, South Africa; 3 Center for Population and Development Studies, Harvard University, Cambridge, MA, USA and; 4 INDEPTH Network, Accra, Ghana

Recent editorials, articles and monographs emphasize that populations of low- and middle-income countries (LMIC) are ageing and that this, in part, is driving the escalation in non-communicable disease (NCD) burden in these countries. Certainly, projections make clear the steep rise anticipated in older populations across South America, Asia and sub-Saharan Africa: those aged 60 years and older will make up 13.1% of the total population of these regions by 2030, and 19.2% by 2050. Indeed, long-term cohort data from rural South Africa show an increase in the proportion of older persons aged 50 years and over from under 10% in 1994 to over 12% in 2014. This phenomenon—of rapid population ageing and rising chronic disease burden with changing dependency arrangements—speaks to the aptness of the sustainable development paradigm and the essential need to incorporate a life course approach into epidemiology and demographic risk perspectives. Adults arrive at older ages having experienced a cumulative set of exposures, starting ‘in utero’, then in early childhood and continuing right up to their later years. Life course perspectives will enable us to understand not only current cohorts reaching older ages in LMICs, but also to predict risks facing the upcoming cohorts, now in childhood, adolescence or early adulthood.

The ageing picture in LMICs is complex, notably in southern and east Africa where the full force of the AIDS pandemic—while fuelling an interacting TB epidemic—is colliding head-on with a rapidly unfolding epidemic of cardiovascular and metabolic disease risk. In parallel, the sizeable sub-group of youth and adults on long-term antiretroviral therapy (ART) is rising rapidly. The social dynamics of family constellations which are impacted on not only by health and illness but also by pervasive labour migration (and forced removals in South Africa under apartheid), reinforce the need to attend to life course contexts. As exposures in one period of life change, so too will population health outcomes from one generation to the next. Evidence on the evolving patterns of behaviour, disease and mortality, their precursors and modifiers, and the consequences for population health and well-being remains sparse. Similarly, evidence on which to build and adapt the health and social systems best suited to respond to these challenges is largely unavailable, contributing to a policy vacuum.

Older adults in sub-Saharan Africa today play essential roles raising not only their own children but also the generation after. A devastating result of the AIDS pandemic has been extensive mortality in the parental generation, leaving surviving grandparents to care for growing children and maintain the social and financial integrity of households. In South Africa, at the epicentre of the pandemic with (conservatively) 26.4% of HIV/AIDS cases in sub-Saharan Africa, amounting to 18.4% globally (UNAIDS, 2015)^1^, levels of orphanhood and fostering are high. Women over 60 years in receipt of pension income tend to foster three times as many children as those households without a female pensioner.^2^ These monies are used to offset food insecurity, improve school attendance and contribute to overall household welfare (pensioners’ reported quality of life improves demonstrably once pension-eligible age is reached).^3^^,^^4^ The roles played by these older adults will have an impact on the life course trajectories of those now in infancy and childhood in ways we barely understand. Such family formations in LMICs speak to the importance of gaining a clearer grasp of both cognitive and functional abilities of older people, as well as the impacts these will have over the life course for younger generations. Intergenerational transmission of disadvantage, stemming from the limited educational opportunities open to the older population (such as those who grew up under apartheid), may spill over to younger generations unless major efforts are made. Building on a deeper understanding of such life course trajectories will enable countries to set up programmes that support personal development and productivity and maintain function of older adults and their younger families—work that in LMICs is in its infancy.

Much remains to be done to assess frailty and quality of life of older persons in LMICs, and to determine how these states are influenced by life course exposures and other experiences in response to complex influences. Comparative research in African and Asian health and demographic surveillance system sites (HDSS), in partnership with WHO-SAGE, found that ‘pain’ and experience of ‘sleep/energy’ were domains that correlated consistently with overall reported health status across cultures and settings.^5^ Prospective follow-up suggested that poor self-reported health, in older women especially, correlates with subsequent mortality in the near -term.^6^^,^^7^ This predictor of pending catastrophe speaks to the importance of timely interventions to offset unnecessary loss which carries such ramifications for family well-being.

Life course approaches which rest on multiple birth cohorts, intergenerational family studies and prospective studies, especially those building on community platforms such as HDSSs, offer opportunities that could be exploited more effectively and contribute to a ‘thicker’, policy-oriented evidence base. The growing number and essential role of elders in LMIC society; the resulting increasing burden of NCDs, notably cardiometabolic conditions; the interaction of chronic infection and NCDs in some settings, particularly sub-Saharan Africa; and the social determinants of high-burden conditions all place enormous pressure on health, welfare and social systems. Planning for the present and anticipating the future depend on life course approaches, especially when new exposures appear and older ones fade. Taken together, the case for boosting work on these issues, and augmenting the research resources and data platforms that enable this, is strong. Indeed, this is a requirement if a critical mass of younger scientists, attuned to the issues and with the analytical competencies necessary, is to emerge in LMICs.

At least six major groups or networks of longitudinal studies and research platforms can be highlighted that together constitute a substantial foundation to advance ageing studies from a life course perspective in LMICs. These include: the INDEPTH Network of health and socio-demographic surveillance sites, constituted in 1998, with a presence in sub-Saharan Africa, Asia and Oceania and today covering some 3.8 million people of all ages;^8^ the COHORTS group of birth cohort studies convened in 2005 and based on major long-term studies located in Brazil, Guatemala, India, the Philippines and South Africa;^9^ a later generation of ageing studies modelled on the US-based Health and Retirement Study but adapted to Brazil, China (CHARLS), India (LASI), Mexico and South Africa (HAALSI);^10^ and the WHO-led SAGE family of studies initiated in 2002 with a presence in China, Ghana, India, Mexico, Russian Federation and South Africa.^11^ To these must be added the 10:66 group of dementia studies focused on South America, Asia and increasingly west and central Africa;^12^ and the large-scale PURE study addressing the ‘causes of causes’ of cardiometabolic conditions across multiple socio-environmental settings.^13^

These resources can address perplexing questions about: the long-term physical and cognitive consequences of persisting child and adolescent undernutrition in obesogenic environments; the effects of long-term ART on vascular health and cognitive function; the prospect of intergenerational effects from insults experienced by one or both parents during their childhood and adolescence; little explored gene-environment interactions that could bring new understanding to common lifestyle risks; and the resulting implications for welfare systems that remain dependent on traditional extended kinship networks.

The COHORTS group, for example, drawing on birth cohort data across five LMIC settings, highlight the longer-term impact of maternal characteristics (such as age, nutritional status, post-partum depression), growth and weight gain in infancy and early childhood, and household conditions on metabolic disease risk and human capital development in adolescence and adulthood.^14^ Pooled data from COHORTS have illustrated the persisting impact of maternal malnutrition across the generations.^15^ However, there is a dearth of multi-level data that connect individual health and development beyond childhood into adolescence and the younger to mature adult period. Hence we are yet to appreciate how best to establish, support and sustain healthier trajectories early in life, that will compensate for both the additive and antagonistic effects of later developmental stages.

From a strategic perspective, there can be little doubt that innovative policy, service and systems development—founded on evidence of effects across the life span and especially during critical or sensitive periods—is needed to counter the unfamiliar health scenarios unfolding in LMICs today. Often when we consider life course issues, we focus almost exclusively on early child exposures that impact on adult health; however, the originators of this concept also discussed the importance of early adult exposures for later life outcomes. We believe this is an area that may be critical for shaping patterns of population health in LMICs. In early adulthood, while lifestyle patterns evolve, men and women are exposed to opportunities (or a lack thereof) to develop strong labour-force attachments with potential for a long and enriching career. This is also the critical period of family formation and, if social, economic and health challenges are severe, opportunities for solid, emotionally cohesive family formation will be threatened. Life course epidemiology can and should make a central contribution to our understanding of determinants of health for men and women of all ages. This, however, is contingent on national and international investment in the infrastructure, training and capacity required for integrated biomedical, social and community-level research along the life course.
